# Long-term effects of phacoemulsification and intraocular lens implantation in a patient with pathologic myopia and extremely long axial length: A case report

**DOI:** 10.1097/MD.0000000000022081

**Published:** 2020-09-11

**Authors:** Yu Yang, Hao Chen, Jingqi An, Wei Fan

**Affiliations:** Department of Ophthalmology, West China Hospital of Sichuan University, Chengdu, Sichuan Province, China.

**Keywords:** capsular stability, capsular tension rings, cataract surgery, extremely long axial length, pathologic myopia

## Abstract

**Rationale:**

To report a rare case of phacoemulsification cataract surgery and intraocular lens implantation that improved visual acuity and capsular stability in a patient with pathologic myopia and axial length >38 mm.

**Patient concerns:**

A 51-year-old Chinese man with high myopia since childhood who had lost sight in his left eye at the age of 25 due to retinal detachment. He was referred for ophthalmological assessment due to decreased vision in the right eye, in which the best-corrected visual acuity at distance was hand motion.

**Diagnoses:**

The patient was diagnosed with cataract, high myopia, subluxated lens, and loose zonules in the right eye. The left eyeball showed atrophy.

**Interventions:**

The patient underwent uneventful phacoemulsification. An intraocular lens (Sensar AR40M) and capsular tension ring were implanted within the capsular bag. After surgery, the patient was given eye drops containing tobramycin and dexamethasone eye drops for 1 month and eye drops containing 0.1% sodium diclofenac for 2 months.

**Outcomes:**

There were no postoperative complications. During 1-year follow-up, uncorrected visual acuity was 20/80 and the manifest refraction was −2.50DS/−1.00DC∗80, with corrected distance visual acuity of 20/60. Cataract surgery maintained adequate vision for daily living.

**Lessons:**

Implantation of specific lens and capsular tension ring as well as prolonged use of non-steroidal anti-inflammatory drugs may help prevent capsular contraction and posterior capsule opacification in patients with pathologic myopia and extremely long axial length.

## 1 Introduction

High axial myopia is defined as refractive error of more than −8.00 diopters or an axial length longer than 26.5 mm.^[1]^ High axial myopia is associated with numerous anatomical changes in the posterior pole of the globe, including the hallmark posterior staphylomas. Other main pathological changes include scleral thinning, weak zonules, vitreous opacity, retinal, and choroidal atrophy.^[2]^ The deformity of the globe, including posterior staphyloma, may facilitate the development of these pathologies. These pathological changes become more serious with longer axial length, causing more severe visual impairment. There are rare clinical reports of high axial myopia with an axial length >35 mm, but to the best of our knowledge, there have been no clinical reports of patients with an axial length >38 mm.

Cataract surgery in patients with high axial myopia can be challenging due to anatomical changes in the eye globe. Long axial length affects cataract surgeries in several ways, such as loose zonules and fluctuating anterior chamber depth, intraoperative posterior capsular rupture, postoperative retinal tears, postoperative capsular contraction syndrome and unpredictable refractive outcomes.^[3]^ Extremely long axial length may necessitate special measures to ensure uneventful cataract surgery that maintains patient's visual function.

## 2 Case presentation

**Figure 1:**
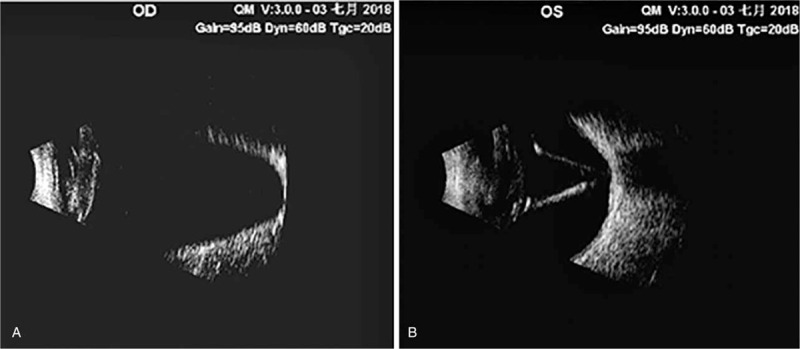
B-scan ultrasonography of both eyes. (A) The right eye showed hallmark posterior staphylomas. (B) The left eye showed retinal detachment and atrophy.

**Figure 2:**
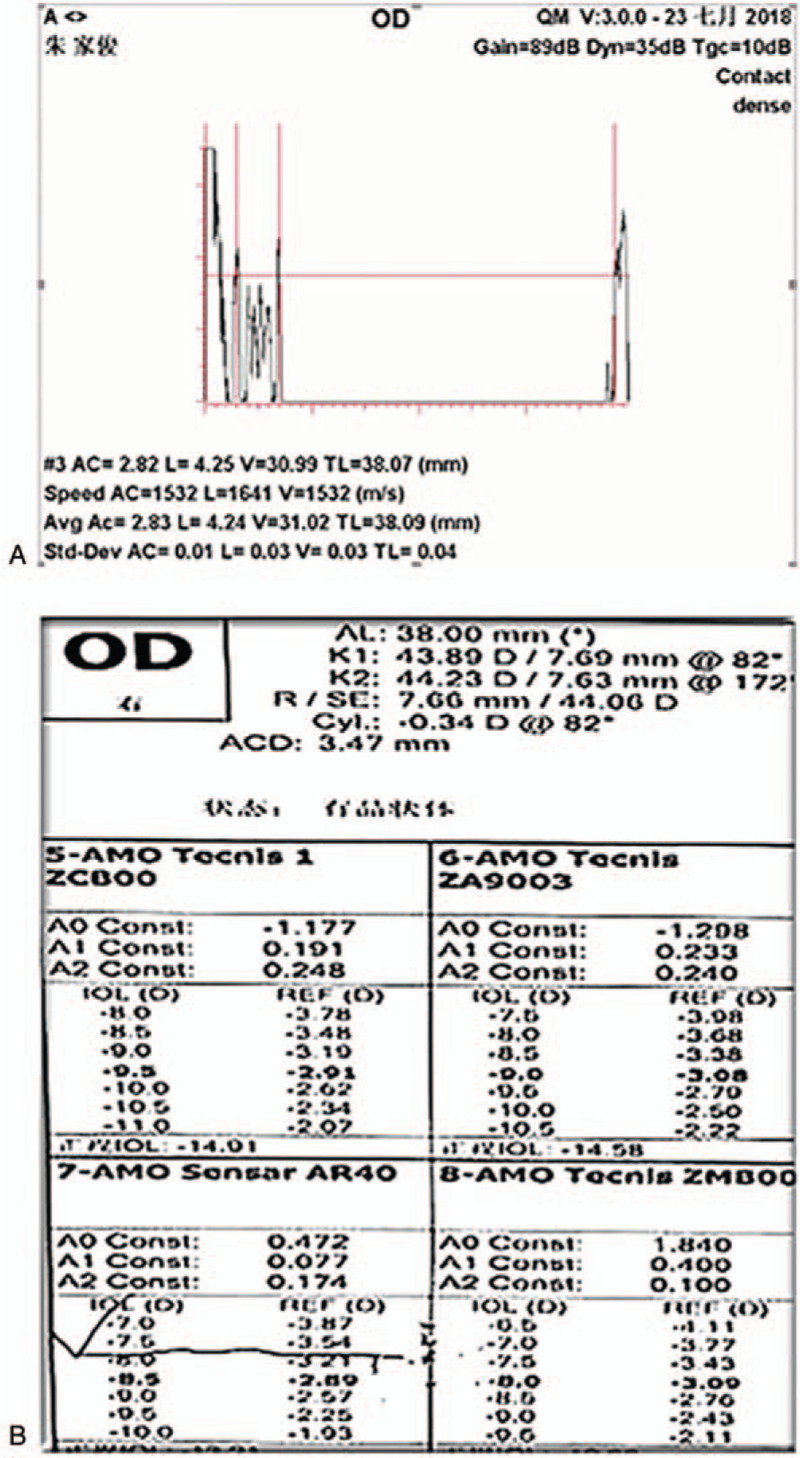
Examination of the right eye. (A) Axial length, measured by A-scan ultrasonography, as 38.09 mm. (B) Optical biometry showed that mean corneal curvature was 44.06 D, and anterior chamber depth was 3.47 mm.

A 51-year-old Chinese male with high myopia since childhood presented at our clinic for ophthalmological assessment due to decreased vision in the right eye. He had been blind in his left eye since the age of 25 due to retinal detachment. His best-corrected visual acuity at distance was hand motion, and the intraocular pressure was 20.3 mm Hg in the right eye. Slit-lamp microscope examination showed iridodonesis and a significant nuclear cataract (C3N4P3). After pupil dilation, a subluxated lens and weak zonules were noted. B-scan ultrasonography showed hallmark posterior staphylomas in the right eye (Fig. 1A), and retinal detachment and atrophy in the left eye (Fig. 1B). Axial length, measured by A-scan ultrasonography, was 38.09 mm (Fig. 2A). Mean corneal curvature was 44.06 D, and anterior chamber depth was 3.47 mm based on optical biometry (IOL Master 500, Carl Zeiss, Oberkochen, Germany) (Fig. 2B). The length of the lens suspensory ligament in the right eye at 270 degrees was 1.71 mm; 270 degrees, 1.25 mm; 180 degrees, 0.99 mm; and 90 degrees, 1.61 mm (Fig. 3 A–D). The density of corneal endothelial cells was 2463 cells/mm^2^.

**Figure 3:**
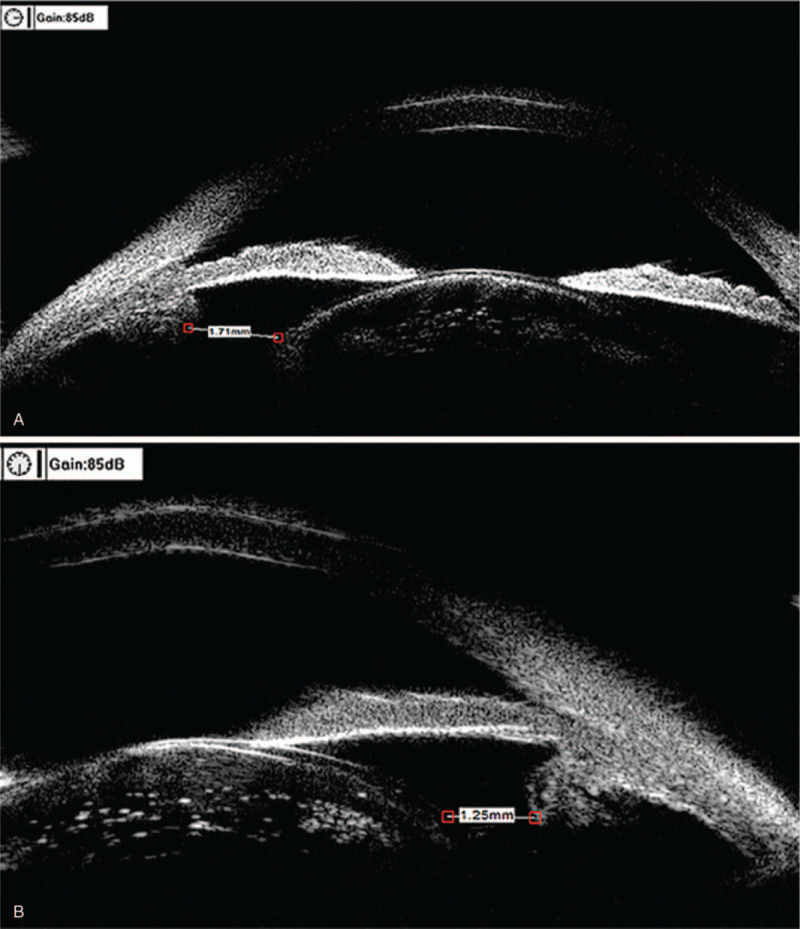
Measurement of the length of the lens suspensory ligament in the (A), 0 degree direction (1.71 mm), (B) 270 degree direction (1.25mm), direction (1.25 mm), (C) 180 degree direction (0.99 mm); and (D) 90 degrees, 1.61mm; direction (1.61 mm).

**Figure 3 (Continued):**
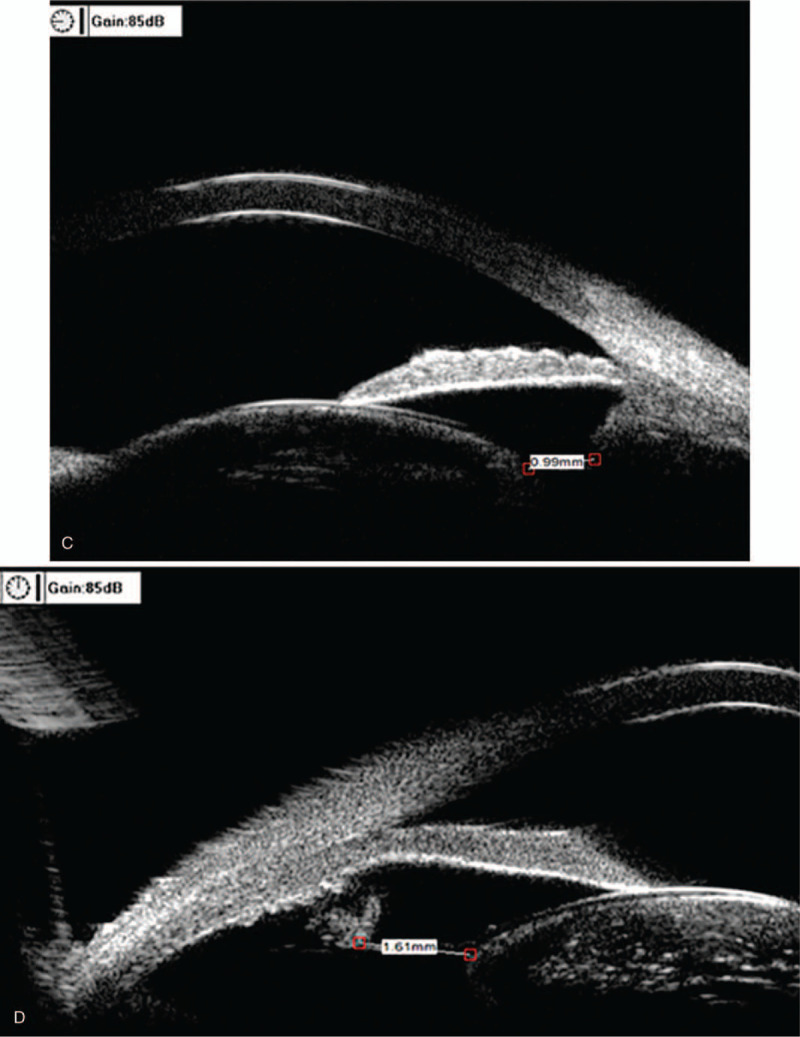
Measurement of the length of the lens suspensory ligament in the (A), 0 degree direction (1.71 mm), (B) 270 degree direction (1.25mm), direction (1.25 mm), (C) 180 degree direction (0.99 mm); and (D) 90 degrees, 1.61mm; direction (1.61 mm).

The patient was diagnosed with cataract, high myopia, subluxated lens in the right eye, and atrophy in the left eye. He underwent uneventful phacoemulsification cataract surgery, during which lower irrigation pressure was applied at an average bottle height of 50 cm (Stellaris Vision Enhancement System, Bausch & Lomb, USA). After the cataract was extracted, a capsular tension ring was implanted to stretch the posterior capsule and stabilize the capsular bag. An intraocular lens (IOL; Sensar AR40M, −7.5 D, reference: −3.54 D) was implanted within the capsular bag. After surgery, the patient was given eye drops containing tobramycin and dexamethasone eye drops (Alcon-Couvreur, USA), which he was instructed to take four times a day for the first week, three times a day for the second week, twice a day for the third week, and once a day for the fourth week. He was also given eye drops containing 0.1% sodium diclofenac (Sinqi Pharmaceutical, China), which he was instructed to take four times a day for 2 months.

**Figure 4:**
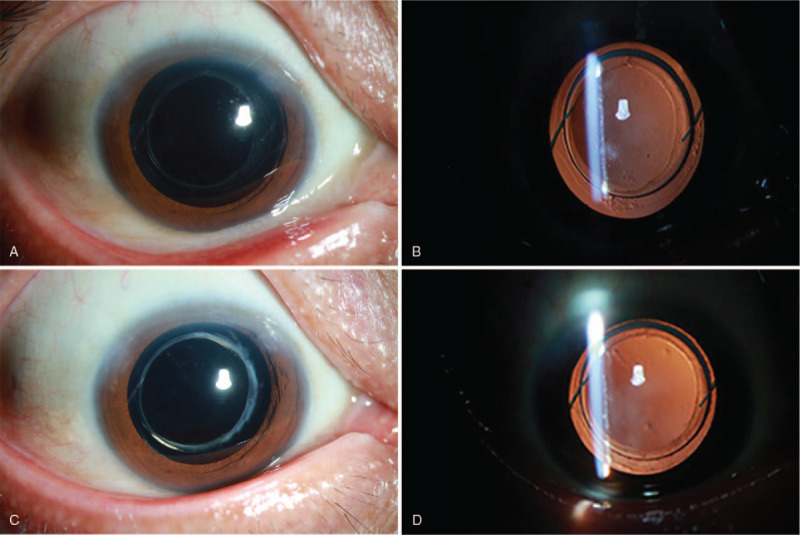
Representative micrographs showing the position of the intraocular lens at (A and B) 3 months and (C and D) 1 year after surgery.

**Figure 5:**
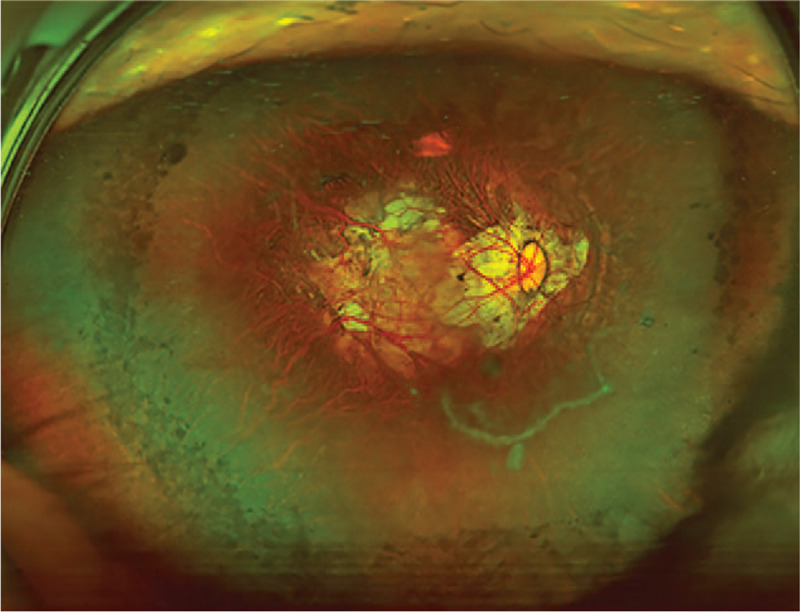
Scanning laser ophthalmoscopy at 1 year after surgery showed tilted discs, peripapillary atrophy, macular pigmentary changes, and large posterior staphylomas in the posterior poles of the right eye.

On postoperative day 1, uncorrected visual acuity (UCVA) was 20/500. Slit-lamp examination showed mild corneal edema and a well-centered IOL with complete capsule overlap. At 3 months after the operation, UCVA had improved to 20/100, and the manifest refraction was −2.75DS/−1.25DC ∗90 with corrected-distance visual acuity of 20/66. The IOL was well centered (Fig. 4A and B). At 1 year after the operation, UCVA had further improved to 20/80 and the manifest refraction was −2.50DS/−1.00DC∗80, with corrected-distance visual acuity of 20/60. The IOL remained centered and overlapped completely with the capsule (Fig. 4C and D). Scanning laser ophthalmoscopy showed tilted discs, peripapillary atrophy, macular pigmentary changes, and large posterior staphylomas in the posterior poles of the right eye (Fig. 5). The patient is being followed up every 3 to 6 months.

## 3 Discussion

This patient presented several challenges that made cataract surgery high-risk: he had suffered retinal detachment in the left eye, the axial length in the right eye was longer than 38 mm, the suspensory ligament of the lens in the right eye was loose and the lens was tilted to the nasal side. In addition, a surge of the anterior chamber and dramatic change in pupil diameter made the surgery more difficult. Therefore, in cataract surgery concerning patients with severe pathologic myopia, most surgeons emphasize maintaining intraocular pressure to avoid rapid pressure fluctuations due to the lack of scleral rigidity.^[3]^ Alternatively, some surgeons remove the lens through the pars plana and vitreous during the surgery,^[4]^ which can stabilize intraocular pressure and allow treatment of any induced retinal tears or lattice degeneration. This second approach is more traumatic, it takes longer and recovery is less predictable. In our case, a corneal incision of 2.0 mm and lower irrigation pressure throughout the surgery were keys to stabilize the anterior chamber until the incision was enlarged to 2.75 mm for IOL implantation. These factors likely helped us achieve uneventful phacoemulsification despite the extremely long axial length and hard nucleus.

Clinical studies have shown that phacoemulsification cataract surgery combined with IOL implantation is safe and effective because the retained posterior capsule can reduce the forward movement of the vitreous and maximize the integrity of the intraocular tissue structure, reducing the risk of retinal detachment.^[5]^ Nevertheless, long-term prognosis may be variable for patients with extremely high axial myopia. The present case suggests that cataract phacoemulsification combined with IOL implantation can indeed be safe and effective even for axial lengths >38 mm, although the procedure is risky and difficult.

Patients with extremely high axial myopia are at high risk of capsule contraction after cataract surgery.^[6]^ The anterior capsular bag can gradually shrink, destabilizing the IOL. High myopic patients, especially those with axial length >27.0 mm, probably have an enlarged capsular bag with weak zonules and tend to develop capsule contraction earlier than emmetropic patients.^[5,7]^ Given the extremely long axial length in our patient, we considered postoperative follow-up slit-lamp examination and dilatation of pupil examinations critical. We did not observe capsule contraction or opacification even at 1 year after surgery. This good outcome may reflect several factors.

One is that during surgery, we were able to achieve continuous curvilinear capsulorhexis and ensure a round, properly sized anterior capsular opening.^[8]^ Another factor may be the mechanical properties of the implanted IOL and capsular tension ring. We used a 3-piece foldable IOL made from hydrophobic optic and polymethyl methacrylate haptic.^[9]^ This polymer haptic shows excellent pressure resistance and can partially resist the shrinkage due to capsule contraction, improving the stability between the IOL and the capsular bag.^[10]^ This lens also features 360-degree rear sharp-edged and front smooth-edged design technology, which can help prevent capsular contraction and posterior capsule opacification (PCO).^[11]^

A third factor in the good outcome may be the implantation of a capsular tension ring, also made of polymethyl methacrylate, due to the subluxated lens and loose zonules. Introduced in 1993, these rings can compensate for zonular defects when treating subluxated cataracts.^[12,13]^ They distribute centrifugal forces among the remaining zonules, while supporting areas of zonular weakness, helping to stabilize the loose lens and allowing the placement of an IOL in the most appropriate position within the capsular bag.^[14]^ The rings can also prevent PCO and postoperative shrinkage of the anterior capsular opening as a result of fibrosis due to capsulorhexis phimosis or capsule contraction syndrome, which could shift the IOL off center.^[15,16]^

Finally, extended postoperative use of nonsteroidal anti-inflammatory drugs (NSAID) may help prevent anterior capsule contraction and PCO in high myopic cataract patients. High myopia has long been considered as an inflammation-related disease, and high myopic eyes are thought to have a proinflammatory internal microenvironment.^[17]^ The anterior chamber of high myopic cataract eyes is in a proinflammatory state that may predispose to inflammation-related complications such as fibrotic capsular contraction syndrome after cataract surgery.^[18]^ Postoperative NSAID solutions can effectively prevent anterior capsule contraction and PCO.^[19]^ In our case, the patient used 0.1% sodium diclofenac ophthalmic solution for two months postoperatively under close follow-up, without adverse reactions. We are convinced that this contributed significantly to the patient's good outcomes after cataract surgery.

Our experience in this case may help clinicians stabilize the postoperative capsular bag in patients with pathologic myopia and extremely long axial length. Prospective studies are needed to optimize procedures and outcomes for such patients.

## Author contributions

**Conceptualization:** Yu Yang, Wei Fan.

**Data curation:** Yu Yang, Jingqi An.

**Investigation:** Hao Chen.

**Methodology:** Yu Yang, Wei Fan.

**Writing original draft:** Yu Yang.

**Writing review & editing:** Wei Fan.
